# Temporal-spatial analysis of mortality from cardiovascular diseases in the State of Ceará, Brazil, between 2009-2019

**DOI:** 10.1590/1980-549720230060

**Published:** 2023-12-11

**Authors:** Aldino Barbosa dos Santos, Adriana de Moraes Bezerra, Lucas Dias Soares Machado, Naanda Kaanna Matos de Souza, Vera Lúcia Mendes de Paula Pessoa

**Affiliations:** I Escola de Saúde Pública do Ceará – Fortaleza (CE), Brazil.; II Universidade Regional do Cariri – Iguatu (CE), Brazil.; III Universidade Federal do Ceará – Fortaleza (CE), Brazil.; IV Universidade Estadual do Ceará – Fortaleza (CE), Brazil.

**Keywords:** Cardiovascular diseases. Mortality. Spatial analysis. Ecological studies. Epidemiology, Doenças cardiovasculares, Mortalidade, Análise espacial, Estudos ecológicos, Epidemiologia

## Abstract

**Objective:**

To analyze the spatial distribution of mortality from cardiovascular diseases in the municipalities of the state of Ceará, Brazil, between 2009-2019.

**Methods:**

This is an ecological study with a spatial focus on the state of Ceará, considering the period from 2009 to 2019. Death data from the Brazilian Mortality Information System and population data from the Brazilian Institute of Geography and Statistics were used to calculate crude and standardized mortality rates from cardiovascular diseases. Temporal analysis was carried out using the Joinpoint Regression Program 4.9.0 software and spatial analysis of the municipalities’ average mortality. The values were smoothed by the local empirical Bayesian method using QGIS 3.16. For spatial clusters, the Global and Local Moran Index was used through Moran Map and LISA Map, with analyses carried out in TerraView 4.2.2.

**Results:**

A total of 132,145 deaths from cardiovascular diseases were recorded in the period, with an average increase of 3% per year. Higher mortality rates were observed in men, people aged ≥80 years, mixed-race ethnicity/skin color, married, and with lower level of education. There was the formation of clusters of municipalities with high mortality rates in the regions of Vale do Jaguaribe, Sertão Central, Centro Sul, Sertão dos Inhamuns and Serra da Ibiapaba.

**Conclusion:**

This study identified municipalities with high mortality and exposed the need for strategies aligned with the reality and particularities of these locations.

## RESUMO

**Objetivo:**

Analisar a distribuição espacial da mortalidade por doenças cardiovasculares nos municípios do Ceará entre 2009 e 2019.

**Métodos:**

Estudo ecológico com enfoque espacial no estado do Ceará, considerando o período de 2009 a 2019. Utilizaram-se dados de óbito do Sistema de Informação sobre Mortalidade e dados populacionais do Instituto Brasileiro de Geografia e Estatística para cálculos de taxas brutas e padronizadas de mortalidade por doenças cardiovasculares. Realizaram-se a análise temporal, pelo *software* Joinpoint Regression Program 4.9.0, e a espacial da mortalidade média dos municípios. Os valores foram suavizados pelo método *bayesiano* empírico local utilizando-se o QGIS 3.16. Para os aglomerados espaciais, adotou-se o Índice de Moran Global e Local por meio do Moran Map e LISA Map, sendo as análises realizadas no TerraView 4.2.2.

**Resultados:**

Foram registrados 132.145 óbitos por doenças cardiovasculares no período, com crescimento médio de 3% ao ano. Maiores taxas de mortalidade foram observadas em homens, pessoas com idade ≥80 anos, raça/cor parda, casados, com menor escolaridade. Houve formação de aglomerados de municípios com altas taxas de mortalidade nas regiões do Vale do Jaguaribe, Sertão Central, Centro Sul, Sertão dos Inhamuns e Serra da Ibiapaba.

**Conclusão:**

Este estudo identificou os municípios com elevada mortalidade e expôs a necessidade de estratégias alinhadas com a realidade e particularidades desses locais.

## INTRODUCTION

In recent decades, society has undergone a series of demographic, social and economic transformations — which include reduced fertility, increased life expectancy, rapid urbanization, inadequate lifestyles, among others — that have resulted in complex changes in health and disease standards, in which chronic noncommunicable diseases (NCDs) have replaced infectious diseases as the primary causes of morbidity and mortality^1,2^.

Among NCDs, cardiovascular diseases (CVD) predominate as the main cause of morbidity and mortality^3^. Since the 1960s, they have been responsible for a considerable burden of disease in Brazil. Of the 72% of deaths resulting from NCDs, 30% were due to CVD, and the highest percentage of these deaths occurred due to coronary disease (32%), stroke (28%), and heart failure (18%)^4–7^.

These transformations in Brazil did not follow a linear and unidirectional pattern. The country still presents important variations due to demographic and socioeconomic differences^8^. However, although there is no uniformity, CVDs have considerably increased in all states^9^.

To illustrate that, we can mention the state of Ceará, where mortality rates from CVD between 1997 and 2017 considerably increased, highlighting cerebrovascular diseases (34.2 to 54.7/100 thousand inhabitants), hypertensive diseases (5.2 to 22.2/100 thousand inhabitants), and ischemic heart diseases (21.5 to 53.5/100 thousand inhabitants)^10^.

Within this context, knowing the risk factors in different population groups will help the health team to provide effective and individualized assistance, based on the prevention of secondary complications and interventions aimed at preventing potential problems, in addition to preserving the organic stability of patients who suffer from CVD^11^.

Therefore, it is important to record the information produced in different areas covered by the Brazilian Unified Health System (SUS), to enable the collection of this information by the Health Information Systems (*Sistemas de Informação em Saúde* – SIS) and, therefore, be used to produce operational and/or epidemiological indicators^12^.

From this perspective, the importance of epidemiological studies in different age groups that aim to identify risk factors, social determinants and the most vulnerable populations is evident^13^. Therefore, we can infer that the distribution of CVD may appear differently, as the context in which different population groups are inserted is variable, which highlights the need to spatially analyze territorial configurations.

In this sense, this study is relevant, considering that the need to understand the epidemiological and spatial characteristics of cardiovascular diseases in the state of Ceará can provide information to health service managers and professionals to adopt strategies that reduce cardiovascular risks and mortality in different population groups and, therefore, improve the quality of life of these individuals.

Thus, the objective was to analyze the spatial distribution of CVD mortality in the municipalities of Ceará between the years 2009 and 2019.

## METHODS

This is an ecological study of secondary health data analysis focused on temporal-spatial analysis.

Data relating to cases of deaths of individuals due to CVD were obtained from the Brazilian Mortality Information System (*Sistema de Informação sobre Mortalidade* – SIM), of the Department of Informatics of the SUS (*Departamento de Informática do Sistema Único de Saúde* – DATASUS) of the Ministry of Health (*Ministério da Saúde* – MS), via Tabnet, through the site of death and place of occurrence.

Conversely, the population data used as a denominator for calculating mortality rates, considering the reference population, come from the 2022 census base of the Brazilian Institute of Geography and Statistics (IBGE) for that year and estimates by municipality, sex, and age for the other years. The municipalities of the state of Ceará, located in the Northeast Region of Brazil, were taken as the locus of analysis.

To analyze the mortality trend due to cardiovascular diseases in the state of Ceará, the period from 2009 to 2019 was selected to better understand the outcome in this historical series, considering CVD as the main cause of death. For the study population, adult individuals aged 20 years or over were selected, according to data available from DATASUS for adults, who were registered with deaths from diseases of the circulatory system.

For the underlying cause of these deaths, chapter IX of the International Classification of Diseases (ICD-10) was adopted, covering all groups of diseases of the circulatory system (I00 to I99).

The following variables were considered for the analysis: sex; age group; skin color/ethnicity; marital status; level of education; deaths by residence; year of death; place of death; place of occurrence; and cause of death according to the ICD-10 Group, for all groups of diseases of the circulatory system.

Information on the epidemiological profile and frequency of deaths from CVD were compiled using the Microsoft Excel 2019 for Windows software. Thus, the nominal variables were analyzed using the absolute frequency and percentage of occurrence in the study population.

Annual mortality was calculated considering the total number of deaths in the state as the numerator and the population of the state in that year as the denominator, taking 100 thousand inhabitants as a reference for this coefficient. Conversely, mortality in each municipality was calculated based on standardization using the indirect method, as this standardization process projects specific rates on the population under study to determine deaths. Using this method, the average number of cases for the period was used, divided by the population of the central year (2014), multiplied by 100 thousand inhabitants.

Initially, the analysis of the temporal trend in mortality from diseases of the circulatory system was carried out, conducted using inflection point regression. Thus, the annual percent change (APC) of the studied trend was evaluated, with a 95% confidence interval (95%CI) and statistical significance p<0.05^14^.

Subsequently, an analysis of the spatial distribution of mortality from diseases of the circulatory system in Ceará was carried out. Initially, a choropleth map of the average mortality from cardiovascular diseases in the municipalities of Ceará was created. As there is a probability of identifying a heterogeneous pattern between municipalities, municipal values were smoothed using the local empirical Bayesian method. This method weights the value of the municipal rate in relation to bordering municipalities using a spatial proximity matrix.

For identifying spatial clusters, the Global and Local Moran Index was used. The Global Moran Index was used to test the spatial dependence hypothesis. The method identifies spatial autocorrelation and can vary between −1 and +1, where values close to zero indicate the absence of spatial dependence, considering p<0.05 significant. If the dependence hypothesis is accepted, the Local Moran Index (Local Index of Spatial Association — LISA) is used to observe the presence of spatial aggregates, given p<0.05^15^.

The results of the above analyses were demonstrated by the Moran Map and the LISA Map. The Moran Map enabled to graphically visualize the degree of similarity between neighbors, represented by four quadrants: in the first, there are municipalities with high rates and which are close to municipalities with equally high rates (high/high spatial pattern); in the second, there are municipalities that have low rates and which are surrounded by municipalities that also have low rates (low/low spatial pattern). The third quadrant contains the high/low spatial pattern; and the fourth, the low/high spatial pattern. The third and fourth quadrants present areas of epidemiological transition, as they demonstrate different patterns^15^.

Temporal pattern analyses were performed using the Joinpoint Regression Program 4.9.0 software. Spatial analyses were carried out using the TerraView 4.2.2 program. Finally, the thematic maps were created in the QGIS 3.16 program. The data used to compose the research are available from the Internet, free of charge, for consultation. In this sense, there is no possibility of causing physical or moral damage from the perspective of the individual or the community. Therefore, the present study did not need to be approved by the Ethics Committee.

## RESULTS

By analyzing the profile of people from Ceará who died due to a CVD, we found a higher prevalence among people aged 80 years or over (43.08%), men (51.91%), mixed-race (65.29 %), married (42.39%), with low (one to three years of completed education) (30.85%) or no (42.88%) level of education. We also noticed that the majority of deaths occurred within the hospital environment (51.85%), followed by home (41.50%). In Table 1 we elucidate these results.

**Table 1:** Epidemiological profile of individuals who died from cardiovascular diseases. State of Ceará, Brazil, 2009–2019.

Variables		n	%
Age group (in years)			
	20–29	1,285	0.83
	30–39	3,064	1.97
	40–49	7,497	4.82
	50–59	14,593	9.39
	60–69	24,535	15.78
	70–79	37,523	24.13
	≥80	66,983	43.08
Sex			
	Men	80,711	51.91
	Women	74,766	48.09
	Ignored	3	0.00
Skin color/Ethnicity			
	White	42,333	27.23
	Black	5,840	3.76
	Asian	399	0.26
	Mixed-race	101,518	65.29
	Indigenous	243	0.16
	Ignored	5,147	3.31
Marital status			
	Single	27,542	17.71
	Married	65,908	42.39
	Widowed	47,483	30.54
	Legally separated	4,462	2.87
	Other	2,656	1.71
	Ignored	7,429	4.78
Level of education (in completed years of study)			
	None	58,066	42.88
	1–3	41,771	30.85
	4–7	20,476	15.12
	8–11	11,224	8.29
	≥12	3,869	2.86
Place of occurrence			
	Hospital	70,212	51.85
	Other health facilities	3,524	2.60
	Home	56,190	41.50
	Public space	2,202	1.63
	Others	3,206	2.37
	Ignored	72	0.05

Source: Department of Informatics of the Brazilian Unified Health System, 2022.

**Graph 1:**
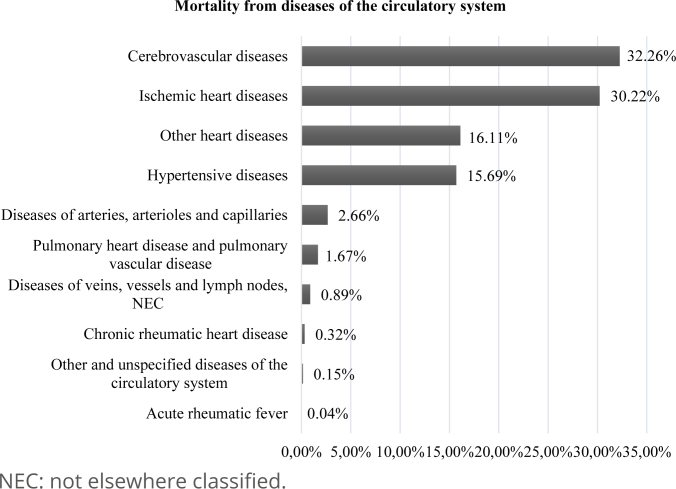
Prevalence of mortality from cardiovascular diseases. State of Ceará, Brazil, 2009–2019.

Source: Department of Informatics of the Brazilian Unified Health System, 2022.

Understanding CVD as a generic term that designates all pathological changes and diseases that affect the heart and/or blood vessels, in Graph 1 we demonstrate that the cerebrovascular diseases and ischemic heart diseases have higher prevalence among the causes of deaths due to CVD, with 32.26 and 30.22%, respectively.

The high prevalence of hypertensive diseases (15.69%) and other forms of heart disease (16.11%) is noteworthy, including pericarditis and other pericardial diseases, endocarditis, non-rheumatic valve disorders, myocarditis, cardiomyopathies, conduction disorders and arrhythmias, cardiac arrest, heart failure, among others.

From 2009 to 2019, 132,145 deaths from diseases of the circulatory system were registered in the state of Ceará. The average mortality for the period was 135.8/100 thousand inhabitants, with the lowest mortality recorded in 2010 (115/100 thousand inhabitants) and the highest in 2019 (155.2/100 thousand inhabitants).

**Figure 1:**
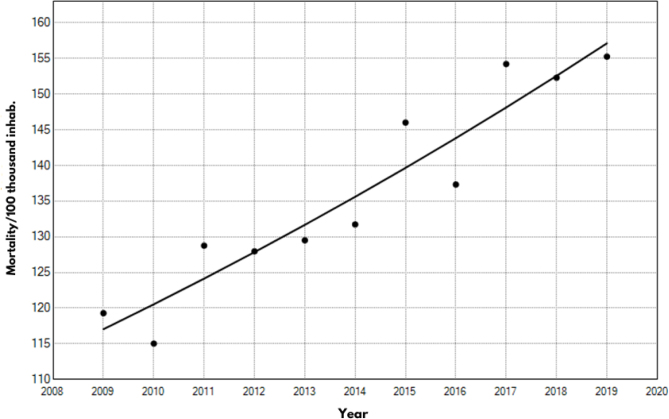
Temporal pattern of mortality from diseases of the circulatory system. State of Ceará, Brazil, 2009–2019.

The analysis of the temporal pattern of mortality in the studied period showed a significant increase of 3% per year (95%CI 2.2–3.8; p<0.001). For this reason, the analysis did not demonstrate the need to insert inflection points. Thus, the trend was explained only by a straight line segment (Figure 1).

**Figure 2:**
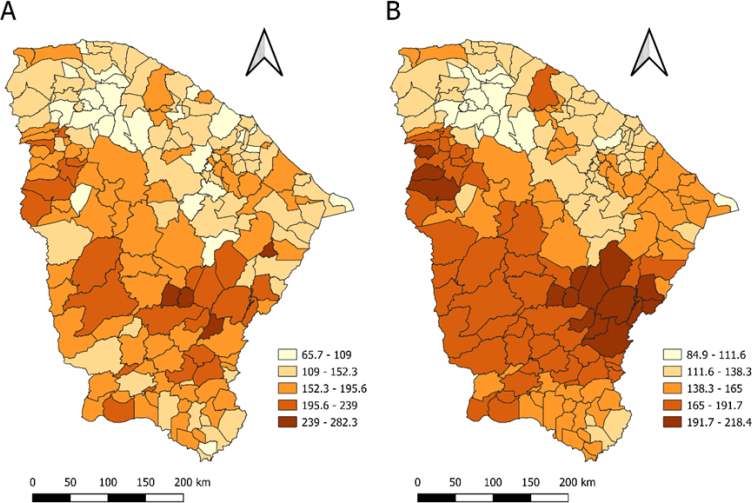
Crude mortality rate (A) and that smoothed by the local empirical Bayesian method (B).

As for the spatial analysis, in Figure 2A we can observe that the municipalities in Ceará had at least a mortality rate of 65.7/100 thousand inhabitants. The municipality of Deputado Irapuan Pinheiro, located in the Sertão Central region, had the highest mortality in the state, with 282/100 thousand inhabitants, followed by Orós in the Central South region, Piquet Carneiro also in the Sertão Central, and São João do Jaguaribe, in the Vale do Jaguaribe region .

By smoothing the crude rates using the local empirical Bayesian method (Figure 2B), we observe a more apparent spatial pattern, with the aggregation of municipalities with higher mortality rates in the Vale do Jaguaribe, Sertão Central, and Centro Sul regions. Moreover, it is worth highlighting municipalities in the Serra da Ibiapaba region with high Bayesian rates.

We identified spatial autocorrelation by using the Global Moran Index (I=0.46; p=0.01), with evidence of positive autocorrelation. Therefore, understanding the statistical significance value of p<0.05, it is considered that the lower this value, the greater the statistical significance and the greater the autocorrelation.

The application of the Local Moran Index enabled us to identify spatial clusters with both high and low equal values. Thus, the high-high pattern was identified mainly in the south of Ceará, highlighting the regions of Vale do Jaguaribe, Sertão Central, Centro Sul, and Sertão dos Inhamuns as well as the Serra da Ibiapaba region. Hence, the municipalities in these regions are similar in that they have high mortality rates due to diseases of the circulatory system.

**Figure 3:**
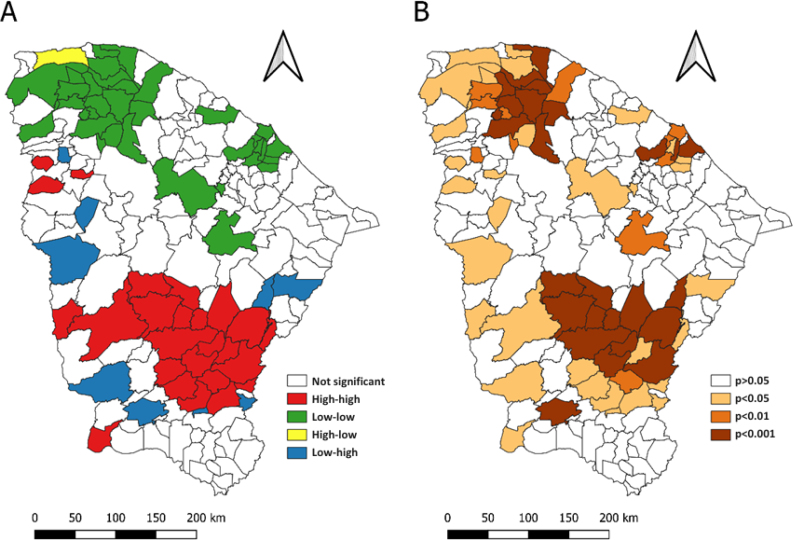
Spatial clusters of mortality due to diseases of the circulatory system in the state of Ceará (A) and statistical significance of the clusters (B).

Conversely, the low-low pattern was identified in the Litoral Norte and Grande Fortaleza regions, indicating similarity between municipalities in these regions for low mortality rates due to these diseases (Figure 3A). In Figure 3B, we can observe the intensity of statistical significance of the clusters, identified using the LISA Map.

Thus, the high mortality rates due to CVD in the state of Ceará are evident, with a temporal pattern of mortality showing significant growth in the last ten years. The sociodemographic variables used for the study showed considerable frequencies with CVD, especially for cerebrovascular diseases, ischemic heart diseases, and hypertensive diseases (Figure 1).

Regarding the spatial analysis with crude indicators, the results demonstrated heterogeneity between mortality rates from diseases of the circulatory system in the state's municipalities. The spatial aggregates found by smoothing these crude mortality rates show municipalities in nearby regions, such as Vale do Jaguaribe, Sertão Central, and Centro Sul, with high mortality rates, which may be the result of similar social, demographic, and/or environmental characteristics.

## DISCUSSION

Mortality statistics are the most used way to understand the health status of a given population and plan the necessary actions to improve their health conditions^16^. Within this context, the results of the present study point to the occurrence of high CVD mortality rates in the state of Ceará. This finding is in line with the study on CVD mortality in the adult population in Brazilian microregions, whose authors state that CVD mortality still occupies a relevant place among the causes of death, despite the vast knowledge of the various risk factors involved in the development of these diseases^17^.

The improvement of public policies carried out in recent decades, as well as the better socioeconomic conditions of a large part of the population, contributed to the reduction in these mortalities. However, when analyzing by federation unit, there is an important regional variation, with less developed states, such as those in the North and Northeast regions, showing a smaller reduction^6^. This heterogeneity in the reduction of these mortalities, together with population growth and aging, also contributes to the high prevalence of deaths from CVD^18^.

The epidemiological patterns of CVD mortality found in the state are more frequent among older ages. This can be justified by the fact that increased longevity naturally leads to a longer period of exposure to risk factors. The need for specific planning to serve this older portion of the population is evident^19^, as significant growth in the number of older people in the state of Ceará is already expected in the coming decades^20^.

Considering the findings, we perceive that CVD mortality is more common in the male population. Men are more exposed to cardiovascular risk factors such as smoking, physical inactivity, excessive alcohol consumption, inadequate eating habits and being overweight. These behaviors express resistance to health care and are associated with sociocultural and institutional factors, which increases contact with risk situations and the challenge of recognizing health and care needs as well as the search for health actions and services^21^.

The higher mortality from CVD also highlighted important differences regarding skin color/ethnicity. Mixed-race/black people are the most vulnerable group, as they have worse metabolic and anthropometric indicators related to cardiovascular diseases such as obesity, dyslipidemia, pre-hypertension/hypertension, and increased circumferences^22^. These findings corroborate the results of our study, as we identified a higher frequency of deaths in these groups, more specifically in mixed-race individuals.

We also identified a higher frequency of CVD deaths in people with lower or no level of education. Such findings are important cardiovascular risk factors, in which CVD mortality rates showed a strong correlation with individuals with less than three years of education. A higher level of education reduces the risk of developing CVD, as it is an important factor for self-health care and can encourage the search for better conditions and quality of life. Thus, the literature shows that people with a lower level of education are more prone to the risk of becoming ill^23^.

When analyzing the types of CVD that led to the death of this population, it is noted that the most prevalent are cerebrovascular diseases, ischemic heart diseases and hypertensive diseases. This result is similar to several studies previously mentioned^4–7,10^, with such diseases being related to higher numbers of hospitalizations and deaths due to diseases of the circulatory system, also showing relevance in the Ceará context.

Research carried out in recent years points to a significant increase in CVD mortality rates in the states of the North and Northeast regions of Brazil^24,25^. This finding is similar to those we found in this study, showing that, despite greater access to health services, advances in health technologies, and the strengthening of public policies in recent years, the number of people who die from a CVD has considerably increased in the last few years in the state of Ceará during the study period.

This can be explained by the fact that, over the years, the mortality information system has been improved with the aim of increasing its coverage and improving the quality of recording the underlying causes of death on the death certificate^24^. Thus, advances in this system enabled to increase the obtainment of deaths and improve the definition of the underlying causes of death in the last decade, especially in the North and Northeast regions.

It is noteworthy that this variation does not only occur between countries, regions and federation units, but also within the state itself, between municipalities. For instance, we can observe in our findings, by the pattern outlined between the years 2009 to 2019 by the spatial distribution of these mortalities in the state of Ceará, that the highest average deaths are found in small municipalities when compared to other municipalities in the state.

In an ecological study on mortality from diseases of the circulatory system and associated factors in municipalities in the state of Minas Gerais (Brazil), researchers identified that small municipalities face difficulties in guaranteeing adequate assistance, as they present challenges ranging from deficient physical care structure to the limitation to qualify and retain health and management professionals^26^.

The same authors also point out that, as the treatment of complications from these diseases is often expensive and depends on specific sophisticated technologies, most small municipalities do not have these services or reference centers. This makes them dependent on the few external locations that have these types of services such as in large cities^26^.

Our study also opens up gaps for other research of this magnitude to spatially analyze CVD deaths over a wider range of years as well as an approach to hospitalization cases to thoroughly evaluate the morbidity and mortality process of these diseases.

It should be noted that this research presents as a limitation the use of secondary data, which can add biases such as underreporting, lack of information, and inconsistencies in filling out the causes of death. Furthe more, the indirect method used for population estimates represents a limitation, since the last census dates back to 2010.

Another aspect worth highlighting is related to the ecological fallacy, due to the effects of data aggregation, the results found for the population may not be repeated at the individual level. It should also be noted that, as the mortality analysis was based only on the underlying cause of death and not on multiple causes, there may be an underestimation of CVD deaths, especially in the population rich in comorbidities.

Moreover, the temporal-spatial analysis enabled to identify priority municipalities among the regions of the state with high CVD mortality. This inequality in mortality rates between municipalities exposes the need for strategies that are in accordance with the reality and particularities of these locations. Thus, this research serves as scientific support for the organization and planning of actions aimed at improving health care in these most vulnerable places, strengthening and qualifying health services, especially Primary Health Care (PHC), to guarantee access and welcoming this population to these services as well as promoting health and preventing problems resulting from these CVDs.
